# Building health care leadership capacity in a developing country via Talent Grooming Programme (TGP): experience sharing from the Ministry of Health Malaysia

**DOI:** 10.1108/LHS-06-2022-0071

**Published:** 2022-11-11

**Authors:** Kun Yun Lee, Munirah Ismail, Pangie Bakit, Norhaniza Zakaria, Nursyahda Zakaria, Norehan Jinah, Delina Kamil, Nor Hayati Ibrahim

**Affiliations:** Centre for Leadership and Professional Development, Institute for Health Management, Shah Alam, Malaysia

**Keywords:** Health care, Health leadership competencies, Health leadership initiatives, Leaders, Case study

## Abstract

**Purpose:**

Formal structured leadership training is increasingly incorporated as a regular fixture in developed nations to produce competent leaders to ensure the provision of quality patient care. However, most low- and middle-income countries (LMICs) rely on one-off external training opportunities for selected individuals as they lack the necessary resources to implement long-term training for a wider pool of potential health care leaders. This case study shares the establishment process of the Talent Grooming Programme for technical health care professionals (TGP), a three-year in-house leadership training programme specially targeted at potential health care leaders in Malaysia.

**Design/methodology/approach:**

This case study aims to share a comprehensive overview of the ideation, conceptualisation and implementation of TGP. The authors also outlined its impact from the individual and organisational perspectives, besides highlighting the lessons learned and recommendations for the way forward.

**Findings:**

TGP set out to deliver experiential learning focusing on formal training, workplace experiences, practical reflection and mentoring by supervisors and other esteemed leaders to fulfil the five competency domains of leadership, organisational governance, communication and relationship, professional values and personal values. The successes and challenges in TGP programme delivery, post-training assessment, outcome evaluation and programme sustainability were outlined.

**Practical implications:**

The authors’ experience in setting up TGP provided valuable learning points for other leadership development programme providers. As for any development programme, a continuous evaluation is vital to ensure its relevance and sustainability.

**Originality/value:**

Certain aspects of TGP establishment can be referenced and modified to adapt to country-specific settings for others to develop similar leadership programme, especially those in LMICs.

## Background

The field of health care leadership has become increasingly complex in the 21st century. Political, socio-economical, technological, legal and environmental issues have driven constant changes in both the public and private health care sectors. With these evolutions, health care personnel, including clinicians, public health physicians and allied health practitioners, are increasingly expected to be skilful in financial, legal and human resource (HR) management, on top of having excellent communication skills and emotional intelligence (Sonnino, 2016). Good integration of leadership practices has been linked with positive impacts at the individual and organisational levels (Hastings *et al.*, 2014; Wong and Cummings, 2007). On the other hand, a low level of leadership skills can compromise staff satisfaction, service performance and ultimately, the quality of care and patient safety (Wong and Cummings, 2007; Mosadeghrad and Ferdosi, 2013; Day *et al.*, 2014; Forsberg *et al.*, 2004).

Traditionally, the training of health care professionals at the graduate level emphasised clinical competencies. Leadership components are minimally incorporated into the curriculum (MacPhail *et al.*, 2015). With the increasing awareness of the importance of health care leadership, many calls for formal leadership development for health care leaders (Sonnino, 2016; McAlearney, 2008). In recent years, structured health care leadership programmes have been rising in developed nations with sufficient resources to support such development. The most notable ones are the Executive Leadership in Academic Medicine and Harvard Leadership Programmes in the USA, as well as the stepwise training by the NHS Leadership Academy in the UK). As more personnel are trained, a pipeline of high-calibre future leaders can be generated to ensure seamless succession planning in the organisation (Morahan *et al.*, 2010).

While the need for leadership development among health care professionals is globally acknowledged, most low- and middle-income countries (LMICs) do not have such dedicated training (Gulati *et al.*, 2021). This is reflected in a recent review in which all studies related to leadership interventions for health-care providers originated from high-income countries (Mianda and Voce, 2018), thus, highlighting the shortage of such programmes in LMICs. Efforts to develop competent health care leaders are challenging, especially in LMICs that lack the necessary resources and support in the form of prominent institutions or agencies (Kakemam *et al.*, 2020). Those promoted as health care leaders rarely have any structured training before the appointment and are expected to obtain the necessary leadership skills via self-learning or one-off training courses (Dorros, 2006; Edmonstone, 2018; Daire and Gilson, 2014), thus, potentially affecting the staff and organisational performance. As a developing country, Malaysia is not spared from the same challenges.

While the best practice from other parts of the world can be taken in as references and guidance, the training and education approaches of leadership skills and competencies must take into account the capacity, culture and needs of the local health care system. In Malaysia, leadership training is mainly incorporated as a minor component in tertiary-level education. Private training companies and consultancy agencies offer a variety of management and leadership courses or seminars at a charge to the public and corporate sectors. On the other hand, governmental agencies such as the National Institute of Public Administration (INTAN), Razak School of Government (RSOG) and the National Performance Management and Delivery Unit conduct short-term leadership courses that mainly cater to civil servants of various government ministries.

**Figure 1.**
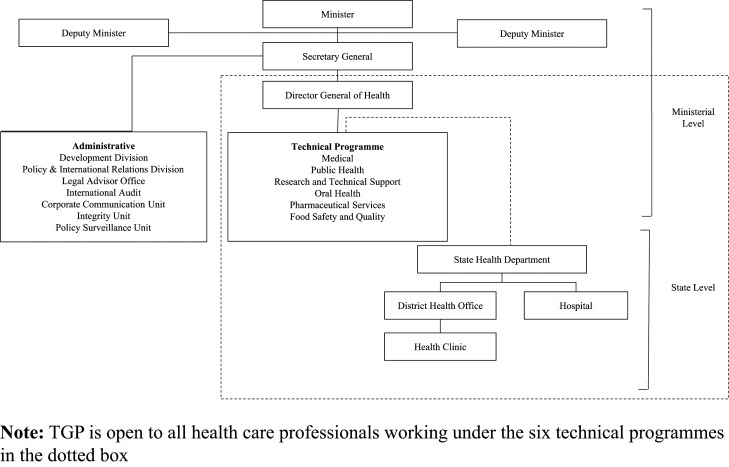
Organisational chart of MOH Malaysia

In the early 2000s, there was no structured and targeted health care leadership training programme in the country. The health care sector in Malaysia is a public-private dichotomous system. The public health care system under the Ministry of Health (MOH) is the main health care service provider. With 255,003 staff Ministry of Health (2022) MOH is one of the biggest ministries in the Malaysian government. Besides the administrative arm, there are six technical programmes (Medical, Public Health, Research and Technical Support [R&TS], Oral Health, Pharmaceutical and Food Safety) involved in the planning, monitoring, execution and delivery of health care (Figure 1).

**Figure 2.**
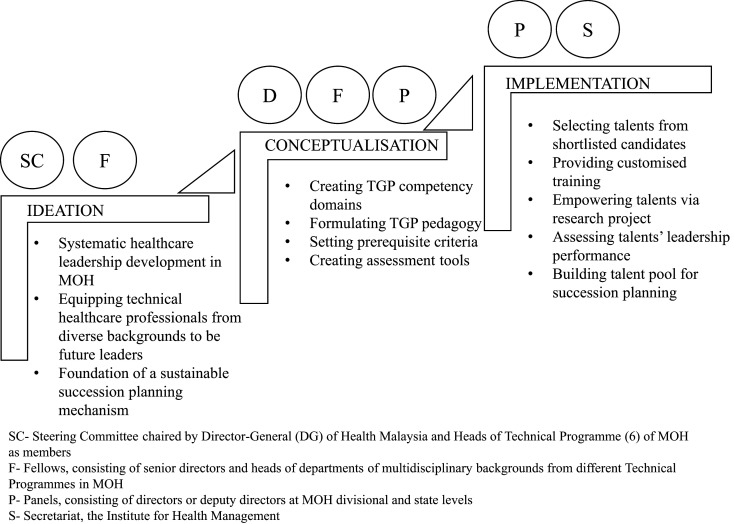
The actors and processes in the ideation, conceptualisation and implementation of TGP in MOH Malaysia

In a broad and multi-level organisation like MOH, close collaboration between professionals from different technical programmes, institutions and regions is vital to overcome organisational silos and achieve a shared vision. Before 2014, there was no formal succession planning and dedicated leadership training for the successors among MOH health care personnel. As individuals ascend the hierarchy ladder in health care organisations, the advancement to leadership positions hinges upon seniority level and professional achievements. Those who rise to supervisory or managerial positions are seldom trained formally in leadership. The Talent Grooming Programme for technical health care professionals (TGP) was the brainchild of the Director-General (DG) of health, who envisioned a structured leadership development programme to identify and use leadership talents at all levels within the MOH for long-term succession planning. To date, besides TGP in MOH, the Ministry of Higher Education is the only other government ministry in Malaysia that had set up its leadership training programme via the Higher Education Leadership Academy to strengthen leadership potential among leaders in Higher Learning Institutions. This case study aimed to discuss the ideation, conceptualisation and implementation of TGP (Figure 2). We also outlined the impact of TGP from the individual and organisational perspectives, besides highlighting the lessons learnt throughout the establishment process before putting forth recommendations for the way forward to enhance the programme.

## Approach

### The ideation behind an in-house leadership development programme for Ministry of Health Malaysia

A systematic review of health care leadership found that many programmes targeted single professions (68%) as compared to multi-professional (22%) and inter-professional (10%) individuals as their learners (Careau *et al.*, 2014). This suggested that the leadership development approach for multi-professional or inter-professional participants has not been explored back then. When TGP was first conceptualised, the emphasis was to develop concerted leadership training for a complex and integrated organisation like MOH. Apart from laying down the foundation of systematic leadership development for MOH, the establishment of TGP was also in response to the call for action by the Public Service Department (PSD) to create a sustainable succession planning mechanism within the Malaysian civil service machinery. In short, the overarching priority of TGP is to be an interdisciplinary and experiential leadership development programme that emphasises collaborative work in diverse teams.

### Conceptualisation of the programme framework for technical health care professionals

TGP Steering Committee was established with its members, including the DG of health and the Heads of all the six Technical Programmes. To distinguish from one-off or short-term leadership training events organised by individual organisations, TGP would be managed centrally by a secretariat, i.e. the Institute for Health Management (IHM) under the R&TS Programme. The TGP Secretariat is tasked with the overall coordination and operation of TGP. The TGP Steering Committee designed the TGP framework and building blocks by referring to leadership development literature and global leadership trend, as well as other ministerial, public and private agencies’ approaches to leadership training and talent management. Experts in the leadership field have described many leadership theories across various sectors. In health care, transformational leadership, clinical leadership, change leadership and transactional leadership are the common competency set expected of leaders. Skills such as growth mindset, problem-solving, role modelling, coaching, communication, networking, empathy, foresight, integrity and financial management are among the highly cited traits of effective health care leaders (Amagoh, 2009; Day *et al.*, 2014; McAlearney, 2008; Macphee *et al.*, 2013). All these were considered by the TGP Steering Committee before designing a three-year programme aimed at equipping health care professionals with the generic leadership competencies aligned with the organisational needs of MOH.

All participants in the programme are known as “talents” to signify their potential as future health care leaders who can be groomed with leadership skills and knowledge from the programme. The potential talents can be technical professionals such as doctors, dentists, nurses, pharmacists and science officers from various levels in MOH, including hospitals, district health offices, state health departments and MOH headquarters. The Steering Committee adopted competency-based training as the core model of leadership development based on the rationale that the training should enhance the individual’s capacity to perform leadership roles based on the competencies required within their organisations (Day, 2000). In terms of pedagogy, TGP set out to incorporate experiential and work-based learning approaches to foster career-long leadership skills that can benefit both individuals and organisations.

Based on these Steering Committee’s guiding concepts, TGP Fellows were handpicked among senior directors and Heads of Departments of multidisciplinary backgrounds to oversee the establishment of TGP Competency Domains and the TGP pedagogy. The TGP Fellows first set out to determine the core competencies deemed critical to the enhancement of leadership capacities. Published literature and existing leadership development programmes were referred to in customising a competency model that fulfilled the goals of TGP.

## Implementation of technical health care professionals

### Technical health care professionals competency domains

**Figure 3.**
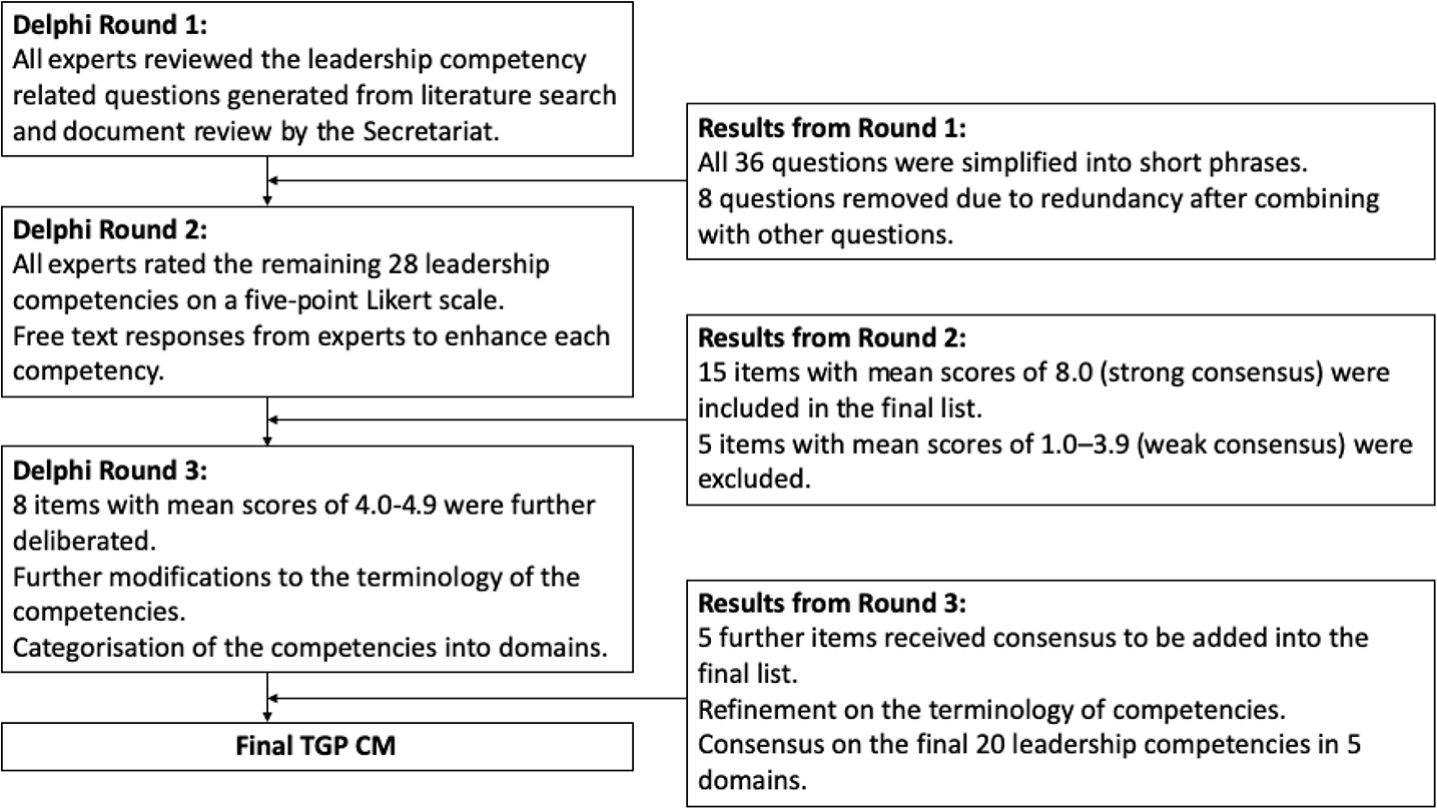
Flowchart of the Delphi process in the development of TGP CM

A competency model can be applied in the process of assessing the gaps, shortlisting the applicants, designing the training and evaluating the outcomes of a leadership training programme (Campion *et al.*, 2011; Liang *et al.*, 2018; Sitzmann *et al.*, 2010). As TGP receives talents with different leadership experiences at various levels of managerial posts in different divisions of MOH, the competency model must be comprehensive to support an appropriate training pedagogy that can cater to the needs of all the talents. For TGP, the TGP Competency Domains formed the basis of the TGP competency matrix (TGP CM). A modified Delphi process was conducted to solicit expert opinions on the development of TGP CM. Two sampling methods, namely, actor types and snowball sampling, were used to identify suitable participants who can provide opinions based on their knowledge, expertise and experiences in health care leadership. For the sampling method of actor type, nine high-ranking officers holding senior positions in MOH were purposively sampled by the DG of health. A further 28 participants recommended by the nine experts appointed were also invited. They included medical and dental officers, allied health professionals, as well as researchers with a special interest or relevant experience in leadership development. Figure 3 shows the process and results from three rounds of Delphi to produce the customised TGP CM for the selection, monitoring and assessment of TGP talents.

**Figure 4.**
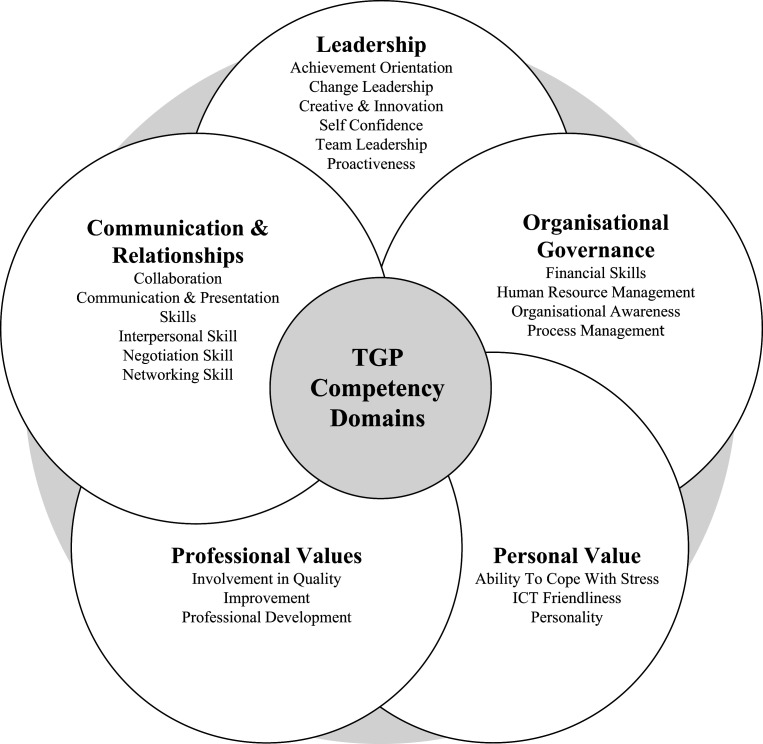
TGP competency domains

The final TGP CM consists of 20 leadership-competency items to be scored from the lowest 1 to the highest score of 5. They are categorised under five domains, namely, leadership, organisational governance and communication and relationship, professional values and personal values (Figure 4). It will be completed at two-time points, i.e. upon joining (pre-TGP CM) and completing TGP (post-TGP CM) by both the talents and their supervisors. The total scores will be categorised into four levels 1: Average, 2: Basic, 3: Intermediate and 4: High for each domain.

### Technical health care professionals pedagogy

For leadership development, educational methods are as important as the curriculum content (Rabin, 2014; West *et al.*, 2015). As aspired by the Steering Committee, the overall pedagogy should emphasise the competency-based goals to be achieved via professional activities and experiential training opportunities (Mansfield, 1996; Thomas *et al.*, 2016). This is aligned with the increasing recognition that leadership development in health care is essentially a practice-based experiential learning process (Raelin, 2016; Edmonstone, 2015). Interventions that are effective in nurturing health care leadership capabilities include discussions, group work and action-learning projects among team members of multi-disciplinary backgrounds (Frich *et al.*, 2015; Rabin, 2014). Therefore, specially-curated courses based on the TGP Competency Domains are delivered by trainers from both government and private sectors to provide a balanced perspective of leadership and management practice. More importantly, lesson plans are developed together with the trainers to customise their suitability to the health care setting. Supplementary 1 shows the training courses in each domain and their respective learning objectives.

However, classroom-based trainer-centric training is not sufficient to groom a well-rounded leader. The majority of TGP training are expected to materialise at the workplace via interactions with colleagues, subordinates and clients under the guidance of a supervisor. Ideally, the supervisor would be senior personnel in the same department who can provide the necessary coaching and mentoring. The positive effect of mentoring and coaching reinforces the learning process and provides a safe environment for emerging leaders to practise their skills (Hawkins and Fontenot, 2010; Daire *et al.*, 2014). The mentorship approach at the workplace provides ongoing support for the talents throughout and even beyond their time in TGP, as echoed by leadership programmes in other countries (Mutale *et al.*, 2017). Furthermore, TGP pedagogy also includes workplace projects that the talents must execute during the training. On-the-job application of leadership skills in any real-world project management following leadership training can be a valuable learning experience to prepare the talents for future leadership roles (Sonnino, 2016; Geerts *et al.*, 2020). TGP projects are undertaken by talents with a focus on creating a positive organisational impact through situational evaluation, problem-solving and innovation. Talents are expected to benefit from the experience of problem identification, project planning, execution and management as well as working in a team to tackle the issue.

With vital concepts of TGP being outlined, the next steps were set in motion to implement the programme, i.e. talent selection for leadership training, knowledge enrichment through partnerships with multiple agencies and experiential sharing sessions by remarkable leaders, as well as post-training assessment.

### Talent intake

TGP application is open to all health care technical professionals from the six technical programmes in MOH (Figure 1). The TGP Selection Panel, which consists of senior officers at MOH headquarters and state-level offices, convenes twice a year to shortlist the new cohort of TGP talents. The selection process of talents begins with the submission of application forms and supporting documents to the TGP Secretariat. All applicants and their referees are also required to complete the TGP CM and an online personality test by PSD. It is highly recommended to incorporate personality tests at the start of leadership development programmes to enlighten the candidates on their characteristics, as well as areas of strength and weaknesses that they should focus on (Sonnino, 2013). The PSD online personality test assesses nine areas, including calmness, cheerfulness, being socially active, expressiveness, sympathy, rational thinking, assertiveness, tolerance and being systematic.

Both assessments provide an overview of the applicants’ personalities with a comprehensive explanation of how the traits influence their daily behaviours, interpersonal interactions and leadership skills. TGP CM results by the applicant and referee are the main evaluation component during the selection process. The panel also takes into consideration other criteria, such as performance reports and continuous professional development scores, both of which form the mandatory annual job assessment for all health care professionals in MOH. The personality report is also used to guide the selection panel. However, there was no structured ranking system in place in the earlier years to determine the weightage of each selection criterion. The final decision is achieved via the consensus of all panel members.

With the first intake of 16 talents in 2014, TGP enrolled 202 talents between 2014 and 2020 from more than 500 applicants (Table 1). They were recruited into 11 cohorts to undergo the three-year programme based on the TGP pedagogy as abovementioned.

### Partnership with external agencies

Besides providing foundational generic leadership training to sharpen the performance of a leader, it is also paramount for future leaders to be exposed to leadership practices outside their organisations (Leigh *et al.*, 2013). Apart from the formal training provided at the IHM, smart partnerships are also forged with other local and international agencies such as RSOG, Genovasi, INTAN and the Royal College of Physicians. These smart partnerships aim to equip TGP talents with better administrative, leadership and public policy insight in their leadership journey. They also learn the best organisational values and culture that can be adopted and adapted in their organisations.

### Inspirational leadership podium

Apart from effective teaching and learning strategies, the key success factors in leadership development also include the exposure of developing leaders to established leaders both within and outside their organisations (Macphee *et al.*, 2013; Leigh *et al.*, 2015). This was the intention behind the TGP Leadership Inspirational Podium, an experience-sharing platform for top leadership figures from diverse backgrounds to impart their wisdom. Since 2014, TGP Inspirational Leadership Podium has been graced by eminent leaders from various sectors, including humanitarian, political, information technology, telecommunication, astronomy and medical tourism (Supplementary 2). The speakers would highlight the challenges encountered in their leadership journey and how they overcame those obstacles. The experience and wisdom serve as a great inspiration for talents, motivating them to use the learning points to improve MOH.

### End-of-training assessment and reflection

**Figure 5.**
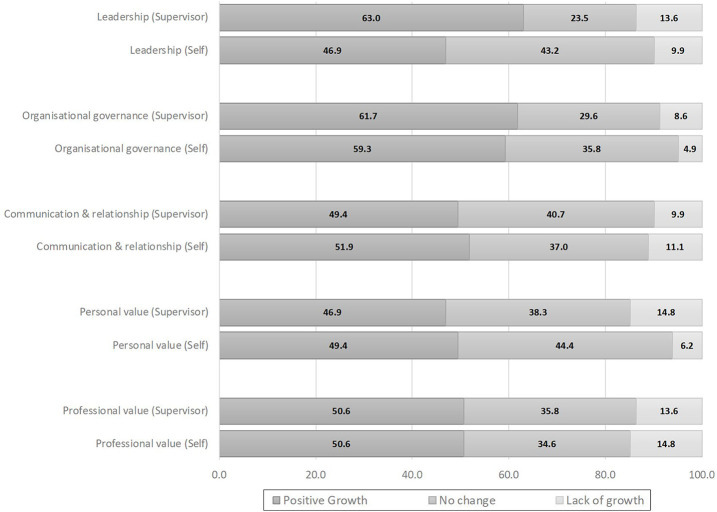
Pre- and post-training TGP CM scores in the five competency domains as assessed by talents and their supervisors (*N* = 81 alumni who have completed the training)

The end-of-programme assessment is conducted to capture the overall results of TGP leadership training. The talents must complete at least 80% of all formal training attendance before the assessment. Based on adult learning theories, an individual’s motivation to learn is driven by the fulfilment of self or others’ expectations and the desire to improve one’s ability to contribute to the organisation (Birkenholz, 1999). TGP assessment is a significant milestone in the journey of the talents that encapsulates individual growth and organisational impact as a result of leadership development. Positive individual growth is defined as an improvement in the level of competencies when comparing post-TGP CM with pre-TGP CM. Figure 5 shows that more than half of the alumni improved in all five domains based on self and supervisor assessment. Among the small proportion with a deterioration in the competency levels, further scrutiny showed that the change was from Level 4 (High) to Level 3 (Intermediate).

In the end-of-training assessment meeting, the talents need to present their TGP project to the Assessment Panel made up of senior officers at MOH headquarters and state levels by elaborating on how the impact of their projects on improving the health care system or work processes in their organisation. Besides polishing their presentation and communication skills, the assessment process also encourages the talents to be confident when seeking stakeholder buy-in for a proposed project recommendation or system change. Table 2 highlights selected TGP projects by the talents that have produced substantial organisational impact, especially the generation of much-needed data and evidence to guide the implementation of intervention at the local level to the policy-making decision at the highest level in MOH.

Apart from the project presentation, talents also reflect upon their learning experience in TGP, especially on the influence of leadership development on their knowledge, behaviours, interaction with others and overall impact on their personal and organisational enhancement. Such reflective activity is vital to consolidate the knowledge and skills taught among the trainees (Rabin, 2014; Amagoh, 2009). Furthermore, relevant and constructive inputs from the talents are incorporated into the betterment of TGP.

## Value

### Technical health care professionals alumni as the future pipeline of Ministry of Health leaders

All talents who successfully pass the end-of-programme assessment will be inducted as TGP Alumni. As a relatively young programme, there have been 81 TGP alumnae between the first assessment conducted in 2016 and 2020. As more talents complete TGP, the pool of potential MOH leaders is gradually expanding. Many alumni in the earlier cohorts have ascended to strategic leadership posts. Out of the 81 alumni, more than one-third (35.8%) of them have been promoted to higher positions after TGP, most of them from the earlier cohorts (1–3). It is projected that the alumni from subsequent cohorts will make up the pipeline of future leaders to be assigned bigger portfolios or higher positions at various levels in MOH. We also observed a good proportion of alumni who were transferred from lower leadership positions in MOH headquarters to higher positions in other facilities (20.6%) as well as from local or state health offices to headquarters (17.5%). This shows the versatility and capability of TGP talents to step into leadership roles in different settings. While not solely attributed to TGP, it is evident that the training and experience gained during TGP play a major role in their career achievement and organisational success.

### Impact of technical health care professionals at individual and organisational levels

Leadership development in health care has been closely associated with many positive impacts. However, not all of such impacts can be objectively measured (Black and Earnest, 2009). More often than not, the benefits are in the form of intangible advantages that are observable but not quantifiable. From an individual-level perspective, many talents have reflected that the most significant benefit was personal growth in terms of personality development. They are more empowered after gaining a better understanding of their personality and leadership styles. Subsequently, this strengthens their interaction and relationship with their subordinates, peers and superiors. Additionally, the knowledge and skills obtained from TGP are assimilated into their practice, thus, enriching their real-life experience in their workplace and propelling them to become better leaders.

More importantly, networking is another highly significant outcome that is difficult to quantify. TGP talents develop a connection with other leaders and like-minded health care professionals via interdisciplinary collaboration opportunities during TGP training or podiums. The great sense of camaraderie among talents motivates sustained synergy and close contact even after their completion, thus opening further opportunities for collaboration for health care professionals from different technical programmes, institutions and regions, breaking down any silos that previously existed. The creation of this community of MOH leaders represents a continuous cycle of support and networking capacity, another great area of strength for TGP. Such long-term networking benefit of the leadership development programme was also highlighted in another study (Sonnino, 2016).

### Strengths of technical health care professionals

TGP was the first leadership development programme designed to cater to the specific target group of health care technical professionals in MOH. It achieves a certain degree of success due to its distinct features. Firstly, in-house delivery of leadership training via TGP is more feasible and cost-effective as it can cater to a larger group of professionals, rather than sending staff individually to courses organised by private trainers that entail higher enrolment costs and staff hours (MacPhail *et al.*, 2015). More importantly, an internally-coordinated programme enables the provision of customised training that applies to the health care practice and fulfils the requirement of the MOH stakeholders (Leigh *et al.*, 2017). Additionally, TGP spans over three years. Long-standing programmes show that leadership training is most effective when it takes place over a certain duration as this enables sufficient time for knowledge immersion, behaviour transformation and the ultimate organisational improvement (MacPhail *et al.*, 2015; Fealy *et al.*, 2015; Miani *et al.*, 2013). Spaced-out training over an extended period is more effective as compared to intensive short courses for leadership development (Arroliga *et al.*, 2014; Savage *et al.*, 2014).

Another unique point of TGP that distinguishes it from other conventional lecture-based leadership training is the incorporation of team-based approaches and work-based learning. By recruiting from every level, division and states, TGP improves the leadership competencies of potential leaders from various technical programmes, job schemes and positions, as well as broadening the interdisciplinary networking and collaboration opportunities for all talents. Furthermore, the commitment and support from the MOH top and ground levels management cemented the success of TGP in grooming potential future leaders. As emphasised in a study, the support by stakeholders at all levels is vital for the success of leadership training (Stoller, 2009). In 2015, TGP was accorded the MOH Star Rating Index, recognition as an innovative strategy that showcased exceptional performance in inculcating a culture of excellence in the national public sector.

### Challenges and lessons learnt

Despite the positive experiences of the participants, TGP was not without challenges in the first few years. For the participants, one of the major challenges lies in course attendance. All talents are expected to fulfil an 80% attendance of the formal training courses at IHM. Unfortunately, this has imposed some pressure on the talents and their workplaces. Some participants reflected on the interruption of service provision to attend training or the need to forgo the training to handle unexpected situations at work. Although a short period of intensive training may be less disruptive, the literature has emphasised that training that is spread out over a longer duration produces better and more sustained leadership impacts (Lacerenza *et al.*, 2017). To overcome this, support from organisation leaders in providing protected time off from work and replacement officers are vital for talents to commit to TGP training. As highlighted in published studies, when the leadership development agenda is taken as a priority, the organisation and its members will actively integrate leadership growth as part of organisational strategy and culture, eventually creating a strong commitment towards leadership development at all levels (McAlearney, 2006; Aarons and Sommerfeld, 2012).

Health care leaders must be trained so that they are capable of meeting emerging needs in a complex health system. This is especially true for LMICs that are facing an increasing double disease burden with the rise of both communicable diseases (CDs) and non-CDs (NCDs). With new challenges and uncertainties surfacing from the constantly evolving health care system, the training offered in TGP must also be updated to match the demands. Furthermore, vital leadership competencies can evolve with time (Fattahi *et al.*, 2020). IHM has continuously reviewed and enhanced the training components based on the latest landscape of health care leadership practice. Finetuning of the topics and teaching pedagogy to fulfil the needs of a wide range of professionals with different lengths and exposure of leadership experience is conducted periodically based on feedback from the participants and trainers. As TGP slowly matures since its initial establishment, it is time for an overall programme review to evaluate its strength, weaknesses and outcomes.

### Way forward

Current and future health care challenges, including an aging population, climate change, emerging and re-emerging diseases, scarcity of resources and growing public expectations, call for more capable leaders to deliver accountable and high-quality care that promises greater health benefits and social values. With the COVID-19 pandemic, fields such as artificial intelligence, big data, Internet of Things are seen as crucial technology advancements that should be embraced by health care leaders (Mahdi, 2019). As such, it is vital to conduct an evaluation of the TGP competency framework and training structure based on the latest understanding regarding the types of leadership capacity required in today’s health care setting. Apart from keeping up with global development, programme evaluation is also imperative to ensure that the future direction of TGP is aligned with the latest projection of the leadership needs of the stakeholders.

As widely acknowledged, the evaluation of the impact of leadership training programmes is challenging for researchers and policymakers alike. Ideally, any assessment of the trained person needs to be carefully planned and measured via pre-post evaluation and 360-degree assessments (Lyons *et al.*, 2020; Seifert *et al.*, 2003; Mianda and Voce, 2018). As TGP is funded by the MOH HR capacity development grant, an objective assessment of the return of investment (ROI) as a measure of organisational outcome is the best way forward in providing evidence to ensure the sustainability of financial resources for TGP in the long-term. As recommended in other published studies (Lyons *et al.*, 2020; Edmonstone, 2013; Avolio *et al.*, 2010), moving forward, the collection of long-term outcome level data of TGP will be incorporated into the programme to obtain the ROI value based on sustainable improvement observed among the talents, in the organisation, and ultimately the service delivery of MOH.

## Conclusion

In summary, this paper outlined the creation of TGP, the first leadership development programme dedicated to TGP in MOH Malaysia. This paper also showcased talents’ upward progression into leadership roles within the organisation and the impact of TGP projects spearheaded by these highly motivated talents. Positive outcomes were visible in the form of personal growth, career advancement and networking opportunities based on the experience sharing of talents. We also proposed how obstacles in the design of a leadership training programme can be overcome with the right resources and expertise. Through TGP, MOH Malaysia has strengthened its commitment to developing the HR capacity and cultivating health care leaders with the right qualities and capabilities to drive high-quality health care service delivery. We hope that this case study will benefit like-minded training providers or administrators who wish to establish a similar leadership development structure.

## Figures and Tables

**Table 1.** Characteristics of TGP talents upon joining the programme

Characteristics	No. (*n*, %)
*Gender*	
Male	64, 31.7
Female	138, 68.3
*Age group*	
<30	10, 5.0
31–35	71, 35.1
36–40	45, 22.3
41–45	53, 26.2
46–50	20, 9.9
>50	3, 1.5
*Technical programme*	
Medical	71, 35.1
Public health	52, 25.7
Pharmaceutical services	27, 13.4
Oral health	26, 12.9
Research and technical support	24, 11.9
Food safety and quality	2, 1.0
*Workplace*	
Headquarters of MOH	46, 22.8
National Institutes of Health	17, 8.4
State Health Offices	34, 16.8
Hospitals	60, 29.7
District Health Offices/primary care	45, 22.3
*Cohort by years	
2014 (Cohort 1)	16
2015 (Cohort 2 and 3)	37
2016 (Cohort 4 and 5)	32
2017 (Cohort 6 and 7)	35
2018 (Cohort 8 and 9)	44
2019 (Cohort 10 and 11)	38

Note: *Only one intake during first year of establishment, Intake suspended in 2020 due to COVID-19 pandemic

**Table 2.** Selected TGP projects and the organisational impact

Selected TGP projects	Organisational impact
1. Making Healthcare Safer: Assessing Patient Safety Culture in Government Hospitals 2. Hand Hygiene Compliance: Human Audits versus Product Usage 3. Improving Care through Lean Implementation in MOH Malaysia 4. Strategy for Needle Stick Injuries Reduction in Health Facilities	Promoting patient safety and good practice culture to improve healthcare quality
1. Oral Health Human Resource Projection towards 2030 2. Work Preparedness of Dental Graduates 3. Stakeholders Perception of Supervision during Housemanship 4. Satisfaction and Perception of Provisionally Registered Pharmacists towards Internship 5. Building Resilient Physicians among junior doctors 6. Workplace Violence Faced by Healthcare Workers	Creating awareness and instilling positive workplace culture. Assisting projection and planning of health human resource
1. Implementation of Hospital Information System for Effective Service Delivery 2. Implementation of Pharmacy Information System in Public Hospitals 3. Patients’ Confidentiality and Privacy Protection in Hospital-based Electronic Medical Record 4. Building Casemix System in Primary Oral Health Care	Using health care digitalisation initiatives for data protection and efficient service delivery
1. Referral Pathways for Chronic Disease Management: Malaysian Public Primary Healthcare Experiences 2. Community Outreach Support for Psychiatric Patients 3. Leading Change in the Management of Periodontal Conditions in Primary Care setting 4. Pre-pregnancy Intervention to reduce the risk of Diabetes and Pre-diabetes	Improving work processes by applying innovation in the provision of universal health care access
1. Regional Consultation on Achieving the Sustainable Goals in the Western Pacific Region 2. Global Surgery Framework Implementation in Public Hospitals	Participating in global agenda and translating new innovation into the local setting
1. Elimination of Measles in Malaysia by 2018: Progress and Challenges 2. Dengue Early Warning and Response System 3. Situational Analysis of NCD Screening Programme in Community 4. Economic Burden of End Stage Renal Disease to Malaysian Health care System	Formulating strategies to mitigate disease burden and its associated economic burden
1. Renewable Distributed Generation in Green Building Rating System for Public Hospitals 2. Health Impacts of Arsenic Exposure in Tap Water in a Local Community	Generating evidence and recommendation in protecting community health and safeguarding environmental sustainability

## Supplementary material

The supplementary material for this article can be found online
